# Lamotrigine-Valproic Acid Interaction Leading to Stevens–Johnson Syndrome

**DOI:** 10.1155/2018/5371854

**Published:** 2018-08-29

**Authors:** Marta Vázquez, Cecilia Maldonado, Natalia Guevara, Andrea Rey, Pietro Fagiolino, Antonella Carozzi, Carlos Azambuja

**Affiliations:** ^1^Department of Pharmaceutical Sciences, Faculty of Chemistry, Universidad de la República, Avenida General Flores 2124, 11800 Montevideo, Uruguay; ^2^Department of Neuropediatrics, Faculty of Medicine, Universidad de la República, Avenida General Flores 2125, 11800 Montevideo, Uruguay; ^3^Genia-Genetics Molecular Laboratory, Bulevar General Artigas 922, 11300 Montevideo, Uruguay

## Abstract

Lamotrigine (LTG) is currently indicated as adjunctive therapy for focal and generalized tonic-clonic seizures and for treatment of bipolar disorder and neuropathic pain. A common concern with LTG in children is the frequency of appearance of skin rash. The intensity of this adverse effect can vary from transient mild rash to Stevens–Johnson syndrome (SJS), which can be fatal mainly when LTG is coadministered with valproic acid (VPA). Hereby, we present the case of an 8-year-old boy who suffered from SJS and other complications two weeks after LTG was added to his VPA treatment in order to control his seizures. VPA is known to decrease LTG clearance via reduced glucuronidation. In this case, the minor elimination pathway of LTG would play a more important role, and the formation of an arene oxide metabolite would be enhanced. As this reactive metabolite is detoxified mainly by enzymatic reactions, involving microsomal epoxide hydrolase and/or GSH-*S*-transferases and these enzymes are polymorphically expressed in humans, arene oxide toxicity is increased when epoxide hydrolase or GSH-*S*-transferases is either defective or inhibited or a depletion of intracellular glutathione levels is taking place. VPA can cause inhibition of epoxide hydrolase enzymes and/or depletion of glutathione levels leading to adverse cutaneous reactions.

## 1. Introduction

Lamotrigine (LTG) is an important anticonvulsant with activity against both focal and generalized onset seizures [1]. It is also effective in the treatment of bipolar disorder [2, 3] and neuropathic pain [4]. However, its use is associated with a significant incidence of adverse cutaneous reactions, mainly when concomitant therapy is administered [5]. Rash is the most common adverse reaction of this antiepileptic agent and the most common reason for treatment discontinuation as it can be life-threatening. Children under polytherapy have a higher risk of this adverse event [6]. Such cutaneous reactions range from mild rashes to more serious conditions such as Stevens–Johnson syndrome (SJS) and toxic epidermal necrolysis [7]. SJS is thought to be a hypersensitivity reaction involving skin and mucous membranes.

LTG shows good absorption after oral administration, and 55% of LTG is bound to plasma proteins mostly to albumin. Its elimination half-life is 25–30 hours under monotherapy [8] and 60 hours approximately when combined with valproic acid (VPA) [9].

**Figure 1:**
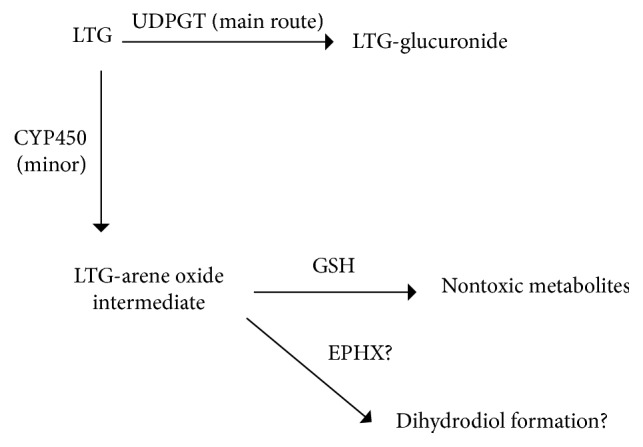
Metabolism pathways of lamotrigine. UDPGT: uridine diphosphate glucuronosyltransferase; EPHX: epoxide hydrolase; GSH: glutathione.

LTG is mainly eliminated by UDP-glucuronosyltransferases leading to the formation of two major metabolites: LTG-N-2 glucuronide and LTG-N-5 glucuronide. A minor metabolism pathway is the formation of the N-2 methyl and the N-2-oxide-LTG [10]. The main route of LTG metabolism does not involve cytochrome P450 enzymes, so according to some authors, the possibility for drug interactions is generally low [11]. However, LTG does have some clinically relevant interactions. As glucuronidation is the main pathway for LTG elimination, anything that inhibits UDP-glucuronosyltransferase enzymes will affect LTG levels, leading LTG metabolism to the minor elimination route in which cytochrome P450 enzymes are involved. So in the absence of the major pathway such as N-glucuronidation, LTG can be bioactivated to an arene oxide [12, 13].  Figure 1 schematizes LTG metabolic pathways.

Arene oxides are of significant toxicological concern because these intermediates are chemically reactive. Arene oxides can be mainly detoxified by rearrangement to arenols, but enzymatic hydration to *trans*-dihydrodiols and/or enzymatic conjugations with GSH also play very important roles. If not effectively detoxified by the aforementioned pathways, an increase in arene oxide will take place, and this reactive metabolite will bind covalently with nucleophilic groups of proteins, DNA, and RNA, leading to cellular damage [14, 15].

VPA is mainly metabolized by the liver, and it is a known inhibitor of UDP-glucuronosyltransferases [16]; according to some investigations [17], there was a significant increase in LTG serum concentrations from 4.67 ± 3.66 to 9.56 ± 5.27 *µ*g/mL by concomitant administration of VPA, indicating a decrease in LTG clearance when VPA was added to the therapy. In this case, the minor elimination pathway of LTG would play a more important role, and the formation of the arene oxide metabolite would be enhanced. As this epoxide appears to be detoxified mainly by enzymatic reactions, involving microsomal epoxide hydrolase (EPHX) and/or GSH-*S*-transferases and these enzymes are polymorphically expressed in humans [18], arene oxide toxicity is increased when EPHX or GSH S-transferases is either defective or inhibited or a depletion of intracellular glutathione levels is taking place. Inhibition of EPHX can occur when VPA is coadministered [19, 20]. High concentrations of VPA can deplete glutathione levels [21]. These two facts could lead to the frequent adverse effects, mainly rash and other cutaneous reactions, reported in the literature [22–24].

Here, we present a case of SJS that occurred after addition of LTG to the VPA regimen.

## 2. Case Report

An 8-year-old boy with chronic encephalopathy secondary to hypoxic ischemic syndrome, with cerebral palsy and symptomatic epilepsy, was admitted to the emergency department of the children's hospital. He had been seizure-free for the past year with an enteric-coated delayed release formulation of VPA (375 mg every 8 hours). Thirty days prior to hospital admission, he was started on LTG 25 mg/day along with VPA, since his seizures were no longer under control with VPA. Two weeks later, the dose was increased (50 mg/day). His morning trough plasma VPA level was measured before LTG was added to the therapy yielding a concentration of 85 mg/L. On admission, he presented macular lesions on the front of the thorax that extend to the back, followed by bilateral eyelid edema and ulcerated lesions at the level of lips, jugal mucosa, and pharynx. He developed erythematosus conjunctivitis with ulcers. He presented skin rash with high fever (39°C) and respiratory failure type I. History revealed that no such lesions occurred earlier and that was the first time such rashes have occurred. Other personal and family history was not relevant. From a dermatologic point of view and based on the history and clinical presentation, a diagnosis of SJS was made. Since the presumptive cause was LTG, the drug was discontinued immediately. Soon after the patient admission, periofocal and ocular involvement worsened. Intravenous immunoglobulin was administered for 48 hours. Mouth care (oral washes with sodium borate) and eye care (tobramycin ophthalmic drops) were also indicated.

From a hemodynamic point of view, four hours after admission, his condition deteriorated and he developed septic shock with peripheral circulatory failure. The patient was admitted to the intensive care unit with intravenous fluids and antibacterial therapy due to skin infection by *Staphylococcus aureus*. In addition to fluid resuscitation, dopamine was administered. Despite the inotropic treatment, the patient's condition did not improve, indicating a septic shock refractory to conventional vasopressor therapy but during treatment with milrinone and norepinephrine for six days (apart from the antibiotics), the septic shock was reversed.

His clinical state steadily improved over the following days. He made an excellent recovery under control seizure and was discharged after twelve days on admission with VPA (375 mg every 8 hours) and oral L-carnitine (2 g/day).

### 2.1. Genotyping Procedure of EPHX

A blood sample (2 mL) of the patient was collected by venipuncture and was refrigerated (4–8°C) until analysis. The Wizard® genomic DNA puriﬁcation kit was used to isolate the genomic DNA from whole blood. Then, it was quantiﬁed by spectrophotometry (260/280 nm) on a NanoQuant-Tecan instrument. EPHX genotype was determined by a real-time polymerase chain reaction using a TaqMan Drug Metabolism Genotyping Assay for rs1051740 and rs2234922.

Two polymorphisms, Tyr113His in exon 3 (SNP rs1051740 T > C) and His139Arg in exon 4 (SNP rs2234922 A > G), have been associated with a decrease or increase in enzyme activity, respectively [18, 25]. 113His/113His or 113His heterozygosity (mutated allele in exon 3) combined with His139/His139 (wild-type allele in exon 4) indicates a decrease in enzyme activity. An increase in activity occurs with 139Arg/139Arg or 139Arg heterozygosity (mutated allele in exon 4) combined with Tyr113/Tyr113 (wild-type allele in exon 3).

The genetic study revealed an increase in EPHX activity (wild-type allele in homozygosity for SNP rs1051740 and heterozygosity for SNP rs2234922).

This study was conducted in accordance with the principles of good clinical practice and the Declaration of Helsinki and was approved by the Ethics Review Committee of the Faculty of Chemistry (Uruguay). Written informed consent of the mother was obtained for the purpose of reporting this case.

## 3. Discussion

Several antiepileptic drugs (AEDs) are used in combination when seizures are poorly controlled. This fact leads to more potential pharmacokinetic and pharmacodynamic interactions in comparison with monotherapy.

When LTG was started, the patient was also receiving VPA for several years, and no adverse effects were recorded during this therapy. However, VPA even at low concentrations [26] is known to decrease LTG clearance leading to higher serum concentrations of LTG which in turn increases the risk of serious rash. This is due, as it was mentioned in Introduction, to VPA's inhibition of glucuronidation pathway [16, 17, 27]. Therefore, concomitant administration of these drugs needs a much slower titration, 25 mg of LTG for the first two weeks and an increase of 25 mg/day for the next two weeks. The starting dosage of LTG in our case was 25 mg/day, and dosage had been increased slowly to 50 mg/day.

Previous work has suggested that the predisposition to such skin reactions evidenced with the use of AEDs is based on a genetic abnormality in the detoxification of reactive metabolites of the drugs [28]. Our research group has been working on this topic with phenytoin; in this case, EPHX is involved in arene oxide detoxification, so we started to investigate EPHX polymorphisms in patients with AED therapy [29, 30]. However, in the case of LTG, both Maggs et al. [12] and Chen et al. [13] research groups conclude that an arene oxide is formed and mainly an enzymatic conjugation with GSH takes place as the glutathione adduct was recovered in the bile. They also showed that human epidermal keratinocytes were capable of forming the GSH conjugate, evidencing that LTG could be bioactivated in skin cells giving a possible explanation for the cutaneous reactions observed with LTG therapy. So in view of their results, LTG can undergo hepatic and nonhepatic bioactivation. Genotyping of GSH S-transferases was not carried out in this patient, so a defective activity of this enzyme cannot be concluded. Nevertheless, VPA and its metabolites (i.e., 4-en-VPA) are likely to produce glutathione depletion or according to some authors, glutathione-*S*-transferases inhibition mainly at high VPA concentrations so detoxification of arene oxide could be impaired, as stated before [31, 32]. VPA is metabolized by three main routes: glucuronidation (50%), *β*-oxidation in the mitochondria using L-carnitine (40%), and *ω*-oxidation (10%); the latter leads to formation of a toxic metabolite, 4-en-VPA [33]. VPA chronic therapy or VPA overdose produces L-carnitine (LCAR) depletion, and this could impair *β*-oxidation leading to the *ω*-oxidation pathway and the formation of toxic metabolites. In our case, predose VPA level was high (85 mg/L). The enteric-coated preparation of VPA administered to our patient would yield important peak-trough fluctuations, so a predose of 85 mg/L could result in a much higher peak concentration. This could have resulted in *β*-oxidation impairment and higher concentrations of toxic metabolites [34–36].

Our patient was discharged with the same dose initially administered of VPA, and LCAR was added. LCAR supplementation restores VPA metabolism to normal routes, increasing *β*-oxidation and thus decreasing *ω*-oxidation and therefore 4-en-VPA formation. Moreover, *β*-oxidation impairment produced perhaps by the initial high concentrations of VPA could have been the cause of hyperammonemia [36, 37], and thus of the occurrence of seizures. That was the reason why LTG was added to the therapy, not taking into consideration that perhaps seizures were the consequence of high levels of ammonia. The introduction of LCAR to VPA therapy would reverse such condition.

Even though there is no current evidence of microsomal EPHX involvement in arene oxide detoxification in the case of LTG and no formation of dihydrodiol was reported in the literature, EPHX participation is chemically possible and cannot be disregarded. Only EPHX polymorphism was studied in this patient, and genetic study yielded an increased activity of the EPHX. On the one hand, the higher catalytic efficiency of EPHX increases the formation of trans‐dihydrodiol, but on the other hand, VPA, as it was stated in Introduction, inhibits the EPHX so perhaps an increase of arene oxide intermediate could have also occurred due to VPA inhibition.

Several studies [38, 39] have determined the association of increased risk of LTG-induced cutaneous reactions with human leukocyte antigen (HLA) genotypes. However, the researchers concluded that further investigation was needed to confirm this association. No HLA genotyping was carried out in this patient in order to infer its implication.

The main limitation of this study is that LCAR, ammonia, and LTG levels were not measured prior admission, and even though no seizures were recorded in the follow-up, once again no VPA, ammonia, and LCAR levels were monitored.

In conclusion, the present case report supports the clinical evidence that a combination of LTG and VPA increases the frequency and severity of skin reactions. VPA not only decreases LTG clearance by competing with the glucuronidation pathway leading LTG metabolism to arene oxide formation, but also inhibits either epoxide hydrolase or GSH-*S*-transferases or depletes glutathione avoiding arene oxide detoxification regardless the polymorphisms of these enzymes. This case also supports the recommendation that antiepileptic levels as well as ammonia levels in patients undergoing chronic VPA treatment should be monitored.
